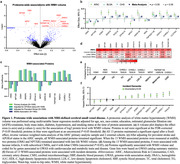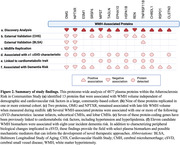# SomaScan plasma proteomics of cSVD

**DOI:** 10.1002/alz.084576

**Published:** 2025-01-09

**Authors:** Gabriela T. Gomez, Liu Shi, Alison E Fohner, Jingsha Chen, Yunju Yang, Myriam Fornage, Michael R Duggan, Zhongsheng Peng, Gulzar Daya, Adrienne Tin, Pascal Schlosser, W.T. Longstreth, Rizwan Kalani, Malveeka Sharma, Bruce M. Psaty, Alejo J Nevado‐Holgado, Noel Buckley, Rebecca F. Gottesman, Pamela L. Lutsey, Clifford R. Jack, Kevin J Sullivan, Thomas H. Mosley, Timothy M. Hughes, Josef Coresh, Keenan A. Walker, Josef Coresh

**Affiliations:** ^1^ Department of Neurology, Johns Hopkins University School of Medicine, Baltimore, MD USA; ^2^ Heptares Therapeutics Ltd, Cambridge UK; ^3^ University of Washington, Seattle, WA USA; ^4^ Johns Hopkins Bloomberg School of Public Health, Baltimore, MD USA; ^5^ The University of Texas Health Science Center at Houston, Houston, TX USA; ^6^ Human Genetics Center, School of Public Health, University of Texas Health Science Center, Houston, TX USA; ^7^ University of Texas Health Science Center at Houston, Houston, TX USA; ^8^ Brown Foundation Institute of Molecular Medicine, McGovern Medical School; School of Public Health, The University of Texas Health Science Center, Houston, TX USA; ^9^ Laboratory of Behavioral Neuroscience, National Institute on Aging, Intramural Research Program, Baltimore, MD USA; ^10^ University of Mississippi Medical Center, Jackson, MS USA; ^11^ Department of Psychiatry, University of Oxford, Oxford UK; ^12^ National Institute of Neurological Disorders & Stroke Intramural Research Program, National Institute of Health, Bethesda, MD USA; ^13^ University of Minnesota School of Public Health, Minneapolis, MN USA; ^14^ Department of Radiology, Mayo Clinic, Rochester, MN USA; ^15^ Wake Forest University School of Medicine, Winston‐Salem, NC USA; ^16^ National Institute of Aging Intramural Research Program, National Institutes of Health, Bethesda, MD USA

## Abstract

**Background:**

Cerebral small vessel disease (cSVD), as defined by neuroimaging characteristics such as white matter hyperintensities (WMHs), cerebral microhemorrhages (CMHs), and lacunar infarcts, is highly prevalent and has been associated with dementia risk and other clinical sequelae. Although risk factors for cSVD have been identified, little is known about the biological processes and molecular mediators that influence cSVD development and progression.

**Methods:**

Within the Atherosclerosis Risk in Communities (ARIC) study, we used SomaScan Multiplexed Proteomic technology to relate 4,877 plasma proteins to concurrently measured MRI‐defined cSVD characteristics, including WMHs, CMHs, and lacunar infarcts, in late‐life (n=1508; mean age: 76). Candidate protein associations with WMH volume were replicated in the Cardiovascular Health Study (n=765; mean age: 73) and Baltimore Longitudinal Study of Aging (n=902; mean age: 66). Cohort‐specific effect estimates were pooled using a fixed‐effect, inverse variance weighted meta‐analysis. Within 4228 non‐demented older adults in ARIC, we examined the association of WMH‐associated proteins with eight‐year dementia risk (641 incident dementia cases) using multivariate Cox proportional hazard models. All multivariate regression models were adjusted for demographic, kidney function, and cardiovascular risk variables.

**Results:**

This proteome‐wide analysis of older adults identified 13 WMH‐associated plasma proteins involved in synaptic function, endothelial integrity, and angiogenesis (Figures 1a, 2a). Nine of these proteins replicated in one or more external cohort (Figures 1b, 2b) and two proteins, oligodendrocyte myelin glycoprotein (OMG) and neuronal pentraxin receptor (NPTXR), remained associated with late‐life WMH volume when measured nearly two decades earlier during midlife (Figures 1c, 2c). Eight of the 13 WMH‐associated proteins were associated with lacunar infarcts, 6 with subcortical CMHs, and 4 with lobar CMHs (Figures 1d, 2d). Although none of the genes coding for WMH‐associated proteins have been nominated by WMH GWAS, 7 of these genes have been previously linked to cardiometabolic traits known to influence cSVD, such as blood pressure and cholesterol (Figures 1e, 2e). Finally, 11 candidate proteins were associated with incident dementia, underscoring their clinical relevance (Figures 1f, 2f).

**Conclusions:**

This large‐scale proteomic analysis within longitudinal community‐based samples identified several plasma proteins, including OMG and NPTXR, as top candidate biomarkers for elevated WMH volume and its clinical manifestations.